# Key Role of Group V Secreted Phospholipase A_2_ in Th2 Cytokine and Dendritic Cell-Driven Airway Hyperresponsiveness and Remodeling

**DOI:** 10.1371/journal.pone.0056172

**Published:** 2013-02-25

**Authors:** William R. Henderson Jr, Xin Ye, Ying Lai, Zhanglin Ni, James G. Bollinger, Ying-Tzang Tien, Emil Y. Chi, Michael H. Gelb

**Affiliations:** 1 Center for Allergy and Inflammation, UW Medicine at South Lake Union, Department of Medicine, University of Washington, Seattle, Washington, United States of America; 2 Department of Chemistry, University of Washington, Seattle, Washington, United States of America; 3 Department of Pathology, University of Washington, Seattle, Washington, United States of America; 4 Department of Biochemistry, University of Washington, Seattle, Washington, United States of America; Leiden University Medical Center, The Netherlands

## Abstract

**Background:**

Previous work has shown that disruption of the gene for group X secreted phospholipase A_2_ (sPLA_2_-X) markedly diminishes airway hyperresponsiveness and remodeling in a mouse asthma model. With the large number of additional sPLA_2_s in the mammalian genome, the involvement of other sPLA_2_s in the asthma model is possible – in particular, the group V sPLA_2_ (sPLA_2_-V) that like sPLA_2_-X is highly active at hydrolyzing membranes of mammalian cells.

**Methodology and Principal Findings:**

The allergen-driven asthma phenotype was significantly reduced in sPLA_2_-V-deficient mice but to a lesser extent than observed previously in sPLA_2_-X-deficient mice. The most striking difference observed between the sPLA_2_-V and sPLA_2_-X knockouts was the significant impairment of the primary immune response to the allergen ovalbumin (OVA) in the sPLA_2_-V^−/−^ mice. The impairment in eicosanoid generation and dendritic cell activation in sPLA2-V^−/−^ mice diminishes Th2 cytokine responses in the airways.

**Conclusions:**

This paper illustrates the diverse roles of sPLA_2_s in the immunopathogenesis of the asthma phenotype and directs attention to developing specific inhibitors of sPLA_2_-V as a potential new therapy to treat asthma and other allergic disorders.

## Introduction

Leukotriene B_4_ (LTB_4_) and the cysteinyl leukotriene (CysLT)s C_4_, D_4_, and E_4_ (LTC_4_, LTD_4_, and LTE_4_) are biologically potent 5-lipoxygenase (5-LO) products of arachidonic acid metabolism [1]. The leukotrienes are important mediators of allergen-induced airway inflammation and remodeling in asthma. They mobilize CD34^+^ pluripotent hematopoeitic stem-cell progenitors from the bone marrow to the bloodstream where they promote adhesion to the endothelium, transmigration into sites of inflammation, and increased survival and activation of leukocytes [1]. Through cross-talk with type 2 helper T cell (Th2) cytokines IL-4, IL-5, and IL-13, the actions of both the Th2 cytokines and leukotrienes are amplified leading to dendritic cell (DC) activation, goblet cell mucus hypersecretion, endothelial cell increased vascular permeability, augmented collagen synthesis by fibroblasts and myofibroblasts, and smooth muscle cell proliferation in the airways [1]. In asthma, other eicosanoids such as the cyclooxygenase (COX) arachidonate product prostaglandin D_2_ (PGD_2_) also contribute to this Th2-driven inflammatory process.

The biosynthesis of eicosanoids is controlled in part by the availability of arachidonic acid, which is thought to be liberated from membrane phospholipids via the action of one or more lipolytic enzymes, most notably phospholipases A_2_ (PLA_2_)s. Mammalian cells contain multiple types of PLA_2_s [2], but it is generally accepted that cytosolic PLA_2_-α (cPLA_2_-α, also known as group IVA PLA_2_) plays a pivotal role in agonist-mediated arachidonate release for the biosynthesis of the eicosanoids. This is based on studies with cPLA_2_-α inhibitors [3]–[6] and studies with cPLA_2_-α-deficient mice [7]–[9]. The mammalian genome also encodes 10 secreted PLA_2_s (sPLA_2_)s. The role of these enzymes in eicosanoid biosynthesis is much less clear. A systematic investigation of the interfacial kinetic and binding properties of the full set of mouse and human sPLA_2_s shows that the group X sPLA_2_ (sPLA_2_-X) stands out as having the highest specific phospholipolysis activity when added to cultured cells [10], [11]. We have recently demonstrated that mice that lack group X sPLA_2_ show a dramatic reduction in parameters of Th2-driven airway inflammation and remodeling [12]. Immunohistochemical studies demonstrate that group X sPLA_2_ is expressed in airway epithelial cells and macrophages in bronchoalveolar lavage (BAL) fluid [12]. Airway hyperreactivity to methacholine challenge, a hallmark asthmatic phenotype, is largely suppressed in the group X sPLA_2_ knockout after ovalbumin (OVA) allergen challenge. Markers of airway remodeling such as occlusion of the airways by mucus and subepithelial deposition of collagen were reduced significantly when sPLA_2_-X was deleted. Although T cell function was unimpaired, sPLA_2_-X-deficiency was characterized by a marked reduction in trafficking of T cells to the allergen-challenged airways in the mouse asthma model [12]. OVA-induced CysLT and PGD_2_ production were near fully blocked in the sPLA_2_-X mouse indicating an important mechanism for the effect of group X sPLA_2_-deficiency. Human group X sPLA_2_ is also found in induced sputum samples in patients with exercise-induced asthma and its levels in BAL fluid correlated with asthma severity [13], supporting a role of this PLA_2_ in human airway inflammation [14].

Group V sPLA_2_ also displays relatively high specific activity when added to mammalian cells in culture that is second to group X sPLA_2_ but well above that of the other mammalian sPLA_2_s [10], [15]. Exogenous addition of nanomolar concentrations of group V sPLA_2_ to neutrophils and eosinophils leads to augmentation of arachidonic acid release and eicosanoid formation [16], [17]. In the case of neutrophils, exogenously added group V sPLA_2_ leads to an activation of cPLA_2_-αsuggesting that these two enzymes work together to maximize arachidonic acid release [16]. In eosinophils, exogenously added group V sPLA_2_ acts without the involvement of the cytosolic PLA_2_ [17]. Disruption of the mouse group V sPLA_2_ leads to a ∼50% reduction in LTC_4_ and prostaglandin E_2_ (PGE_2_) production in peritoneal macrophages that have been stimulated with the fungal-derived agonist opsonized zymosan [18]. In these cells there is also crosstalk between group V sPLA_2_ and cPLA_2_-α. The mechanistic basis for this crosstalk between secreted and cytosolic PLA_2_s remains to be determined. These studies point to the possible role of group V sPLA_2_ in promoting eicosanoid biosynthesis related to inflammation. It should be mentioned that, in general, the study of secreted enzymes with single types of primary cells or cell lines in culture is very different than the study of these enzymes in a whole animal disease model. Secreted enzymes including sPLA_2_ can obviously act on cells different than those that produce them.

Based on these early actions of group V sPLA_2_ and the need to carry out whole animal studies of sPLA_2_s, we investigated the possible role of sPLA_2_-V in mouse asthma models by using sPLA_2_-V-deficient mouse for studies of allergen-induced airway inflammation, hyperresponsiveness, and remodeling.

## Results

### Effect of sPLA_2_-V Deficiency on Acute Asthma Phenotype

**Figure 1:**
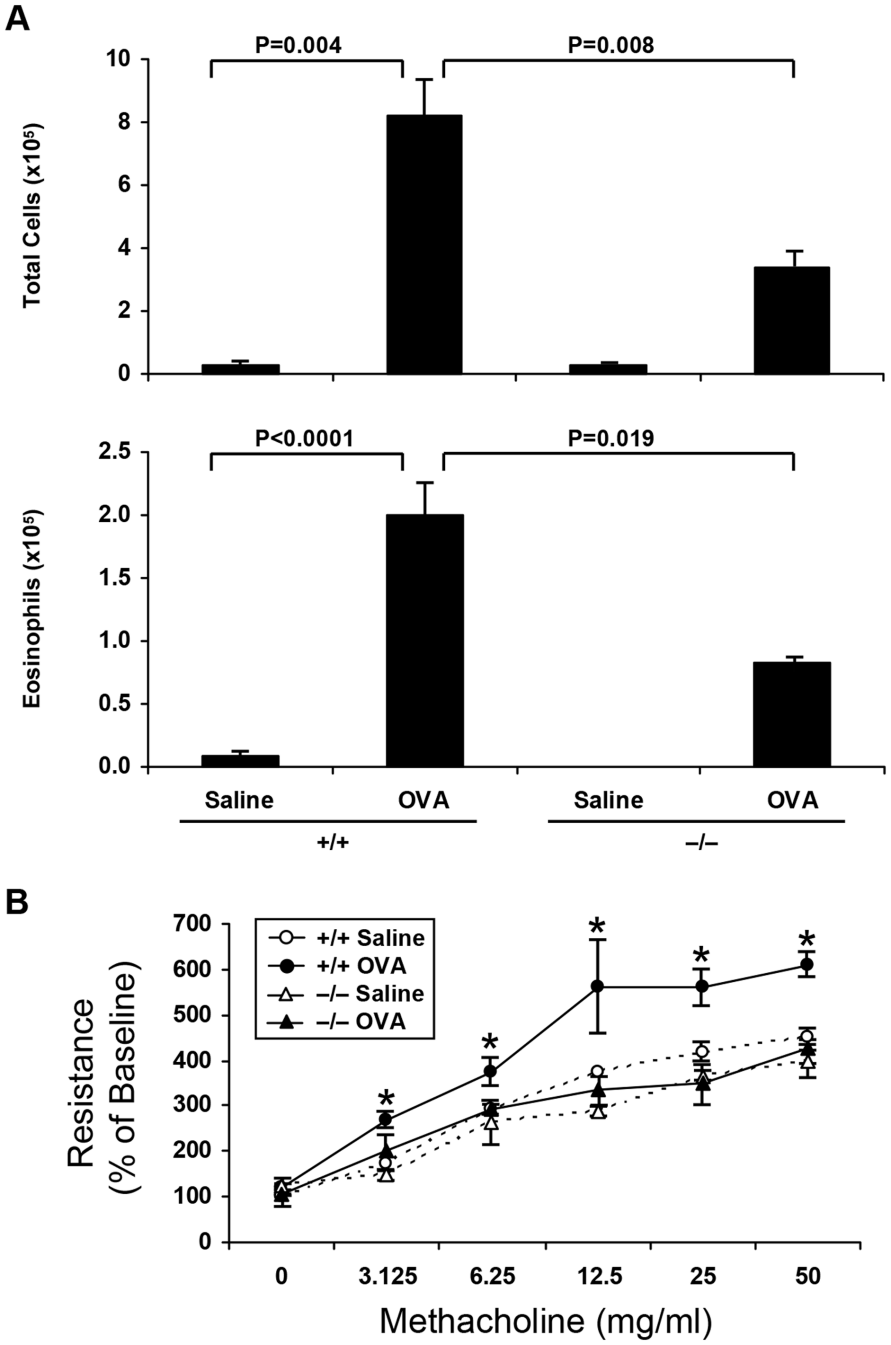
Impaired acute asthma phenotype in OVA-treated sPLA_2_-V^−/−^ mice. **A.** BAL fluid was obtained on d 23 from saline (*Saline*)- and OVA (*OVA*)-treated sPLA_2_-V^+/+^ mice (+/+), and saline (*Saline*)- and OVA (*OVA*)-treated sPLA_2_-V^−/−^ mice (−/−), and the number of total cells and eosinophils was determined (*n* = 4–5, each group). **B.** Airway hyperresponsiveness to aerosolized methacholine (0–50 mg/ml) was determined by invasive plethysmography on d 23 in OVA-sensitized/challenged wild-type (black circles, +/+ *OVA*) and sPLA_2_- V^−/−^ (black triangles, −/− *OVA*) mice in comparison to saline-treated sPLA_2_-V^+/+^ (white circles, +/+ *Saline*) and sPLA_2_-V^−/−^ (white triangles, −/− *Saline*) controls. Lung resistance is shown as the percentage of baseline response to aerosolized normal saline. *P<0.05 versus respective saline-treated sPLA_2_-V^+/+^ or sPLA_2_-V^−/−^ controls (*n* = 4−5, each group).

The effect of sPLA_2_-V-deficiency on allergen-induced inflammatory cell infiltration in the BAL fluid and bronchial hyperresponsiveness was determined in a mouse acute asthma model (**Figure** **1**). OVA-treated sPLA_2_-V^+/+^ mice had a marked increase in both total inflammatory cells and eosinophils recovered in BAL fluid compared with the saline group control (**Figure** **1A**). The number of total inflammatory cells and eosinophils in the BAL fluid of OVA-treated sPLA_2_-V^−/−^ mice was reduced by 59% (*P* = 0.008) and 54% (*P* = 0.019) respectively compared to sPLA_2_-V^+/+^ controls (**Figure** **1A**). The OVA-treated wild-type mice, in comparison to saline controls, had significantly increased responsiveness to aerosolized methacholine as determined by lung resistance (R_L_) (**Figure** **1B**). In contrast, hyperresponsiveness to methacholine after OVA challenge to sPLA_2_-V^−/−^ was similar to that measured in saline-treated sPLA_2_-V^−/−^ mice (**Figure** **1B**).

**Figure 2:**
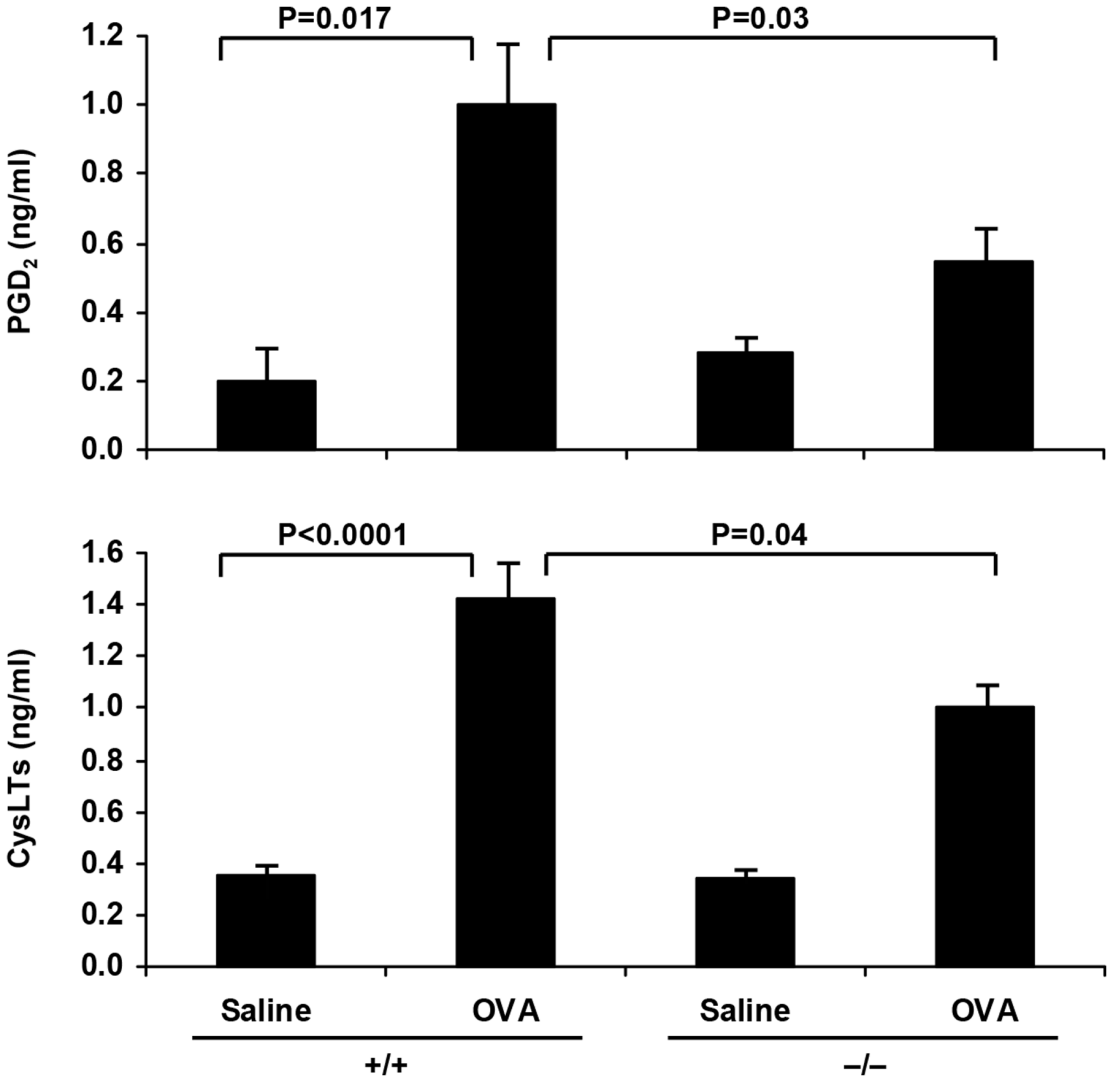
Impaired release of eicosanoids in sPLA_2_-V^−/−^ mice. PGD_2_ (i.e., PGD_2_-MOX) and total CysLT levels were determined in BAL fluid obtained 1 h following the last aerosol OVA challenge on d 23 from saline (*Saline*)- and OVA (*OVA*)-treated sPLA_2_-V^+/+^ (+/+) and sPLA_2_-V^−/−^ (−/−) mice (*n* = 4–5, each group).

The effect of sPLA_2_-V deficiency on levels of eicosanoids derived from arachidonic acid via the cyclooxygenase pathway leading to PGD_2_ and via the 5-LO pathway leading to CysLTs, D_4_, and E_4_ was determined (**Figure** **2**). Since PGD_2_ is unstable, it was converted to its stable methoxime (MOX) derivative prior to measurement. PGD_2_ and CysLTs were significantly increased in the BAL fluid of OVA-treated sPLA_2_-V^+/+^ mice on d 23 in comparison to saline controls (**Figure** **2**). The BAL fluid levels of PGD_2_ and CysLTs of OVA-challenged sPLA_2_-V^−/−^ mice were decreased by 45% (*P* = 0.03) and 30% (*P* = 0.04) respectively compared to the OVA-treated wild-type mice (**Figure** **2**).

### Effect of sPLA_2_-V Deficiency on Chronic Asthma Phenotype

**Figure 3:**
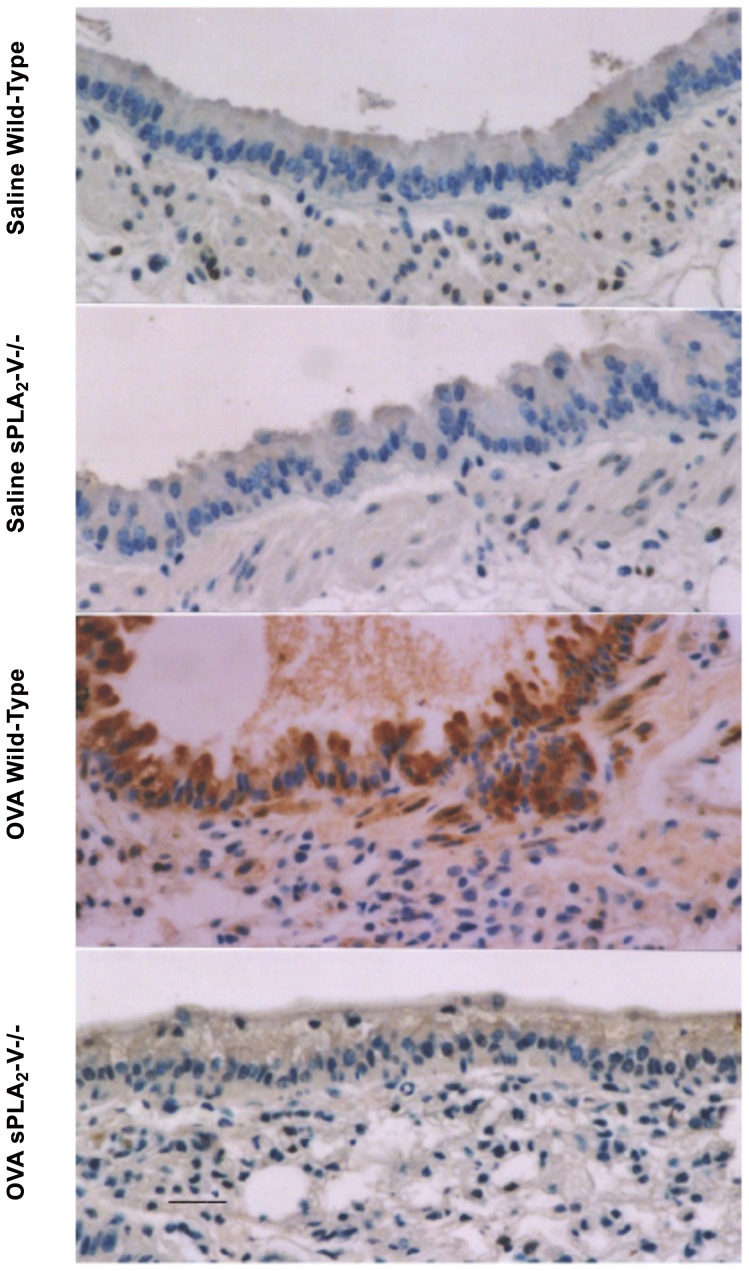
Upregulation of sPLA_2_-V expression in lungs of OVA-treated sPLA_2_-V^+/+^ mice. Lung tissue was obtained on d 76 from sPLA_2_-V^+/+^ and sPLA_2_-V^−/−^ mice treated with either OVA or saline and examined by immunocytochemistry for sPLA_2_-V expression. Scale bar, 50 μm. Micrographs are representative from 4–5 mice per group.

Lung sPLA_2_-V expression was examined on d 76 by immunocytochemistry in wild-type mice and in sPLA_2_-V^−/−^ mice as a control. sPLA_2_-V was undetected in saline-treated sPLA_2_-V^+/+^ controls and in sPLA_2_-V^−/−^ mice after saline or OVA treatment (**Figure** **3**). sPLA_2_-V expression was observed in the airway columnar epithelial cells, airway smooth muscle cells, and mononuclear leukocytes infiltrating the lung interstitium of OVA-treated wild-type mice (**Figure** **3**). sPLA_2_-V was not detected in lungs from OVA-treated wild-type mice when immunocytochemistry was performed with pre-immune serum (not shown).

**Figure 4:**
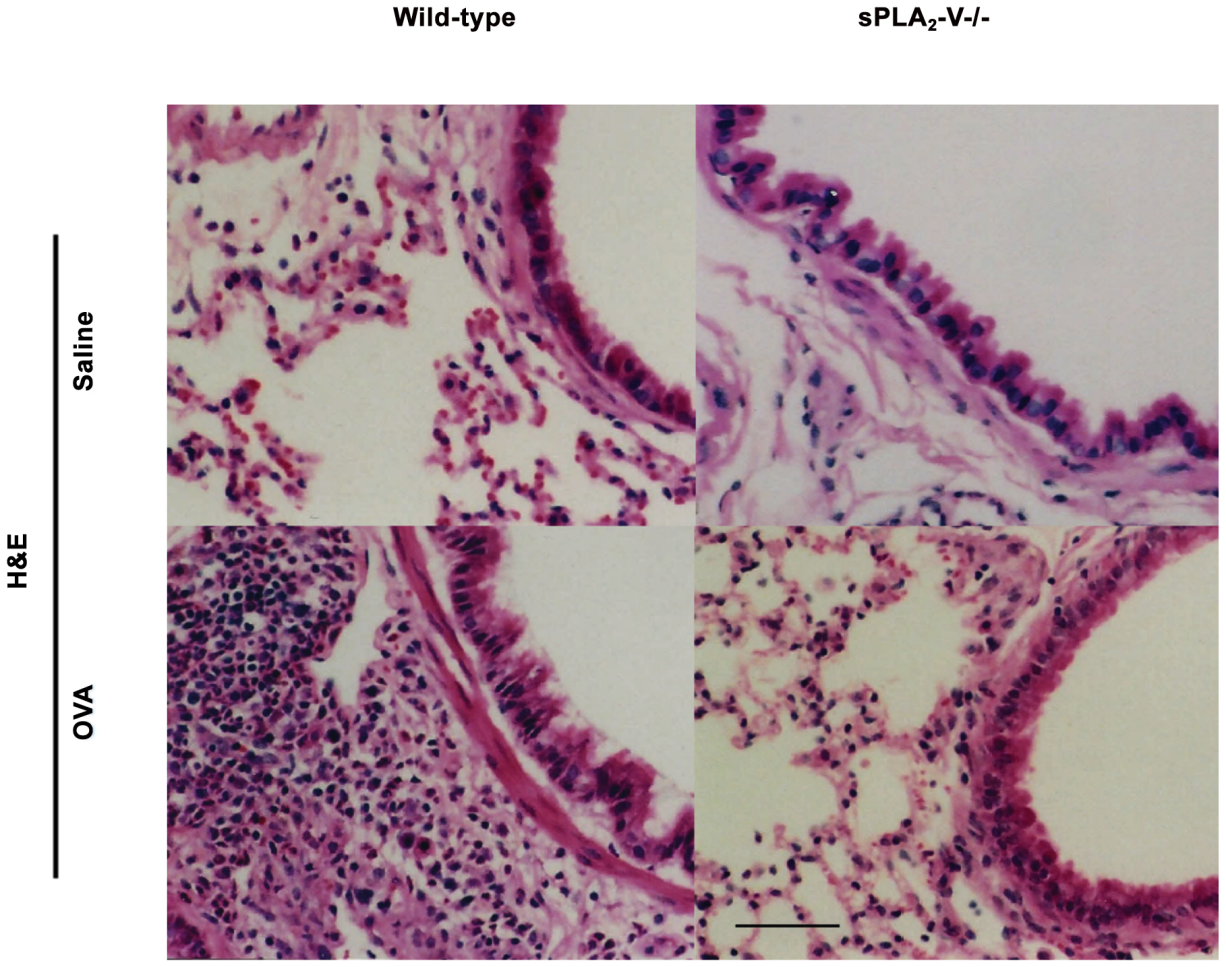
Impaired allergen-induced chronic airway inflammation in sPLA_2_-V^−/−^ mice. Lung tissue was obtained on d 76 from saline- and OVA-treated sPLA_2_-V^+/+^ wild-type mice and sPLA_2_-V^−/−^ mice. Sections were stained with H&E. Scale bar, 100 μm. Micrographs are representative from 4–5 mice per group.

**Figure 5:**
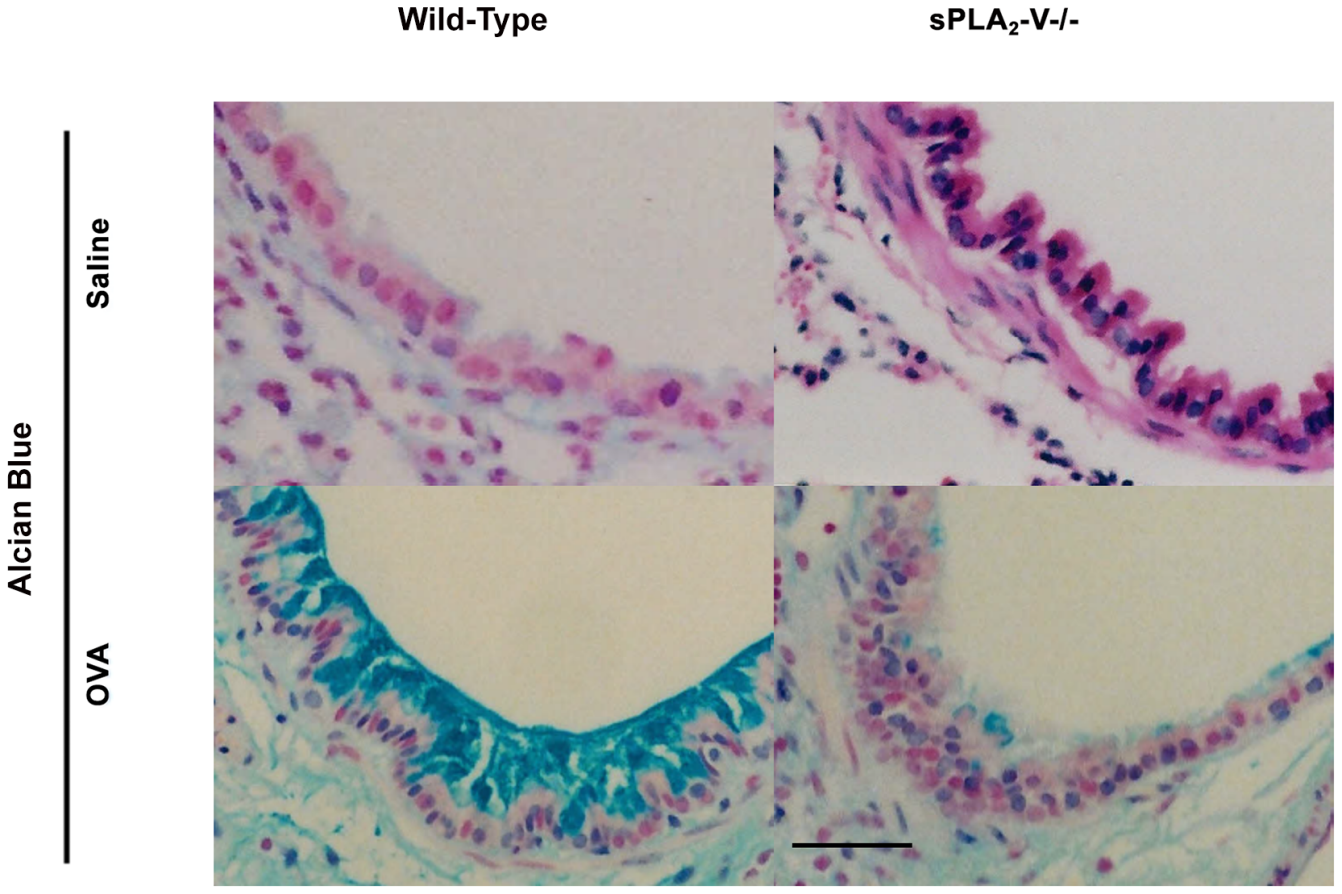
Impaired chronic allergen-induced airway goblet cell metaplasia in sPLA_2_-V^−/−^ mice. Lung tissue was obtained on d 76 from saline- and OVA-treated sPLA_2_-V^+/+^ wild-type mice and sPLA_2_-V^−/−^ mice. Sections were stained with alcian blue. Scale bar, 100 μm. Micrographs are representative from 4–5 mice per group.

**Figure 6:**
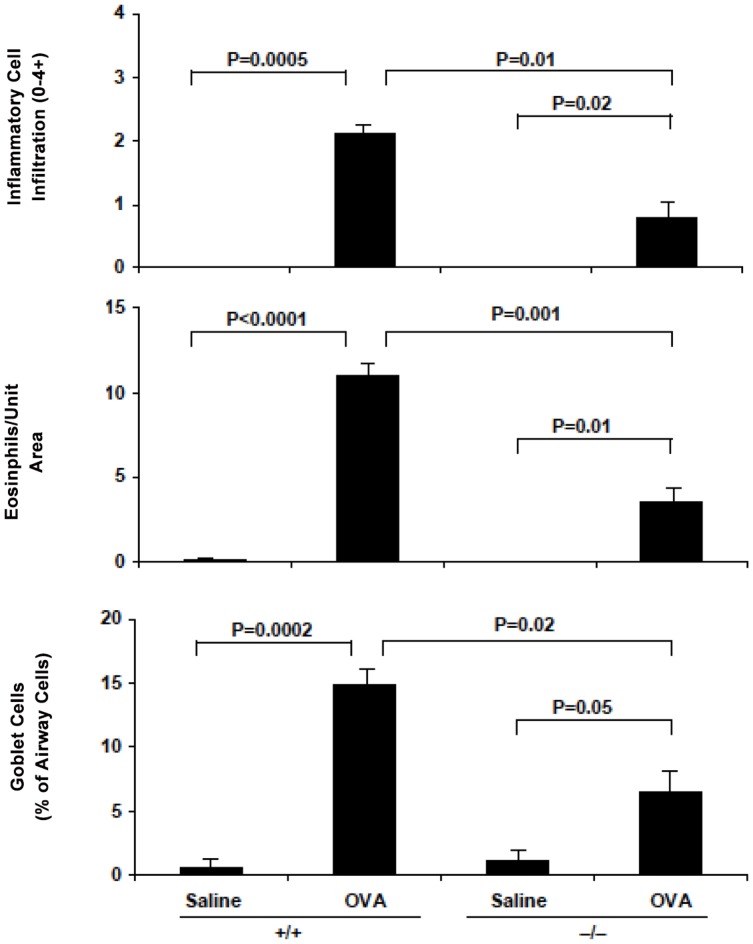
Morphometric analysis of chronic allergen-induced airway inflammation and goblet cell metaplasia in sPLA_2_-V^−/−^ mice. Lung tissue was obtained on d 76 from saline- and OVA-treated sPLA_2_-V^+/+^ wild-type mice and sPLA_2_-V^−/−^ mice. The intensity of the inflammatory cell infiltration (0–4+ scale), number of eosinophils per unit of lung tissue area (2,200 μm^2^), and the number of goblet cells as percentage of total airway epithelial cells positive for mucus glycoproteins were determined by morphometric analysis (*n* = 4–5, each group).

**Figure 7:**
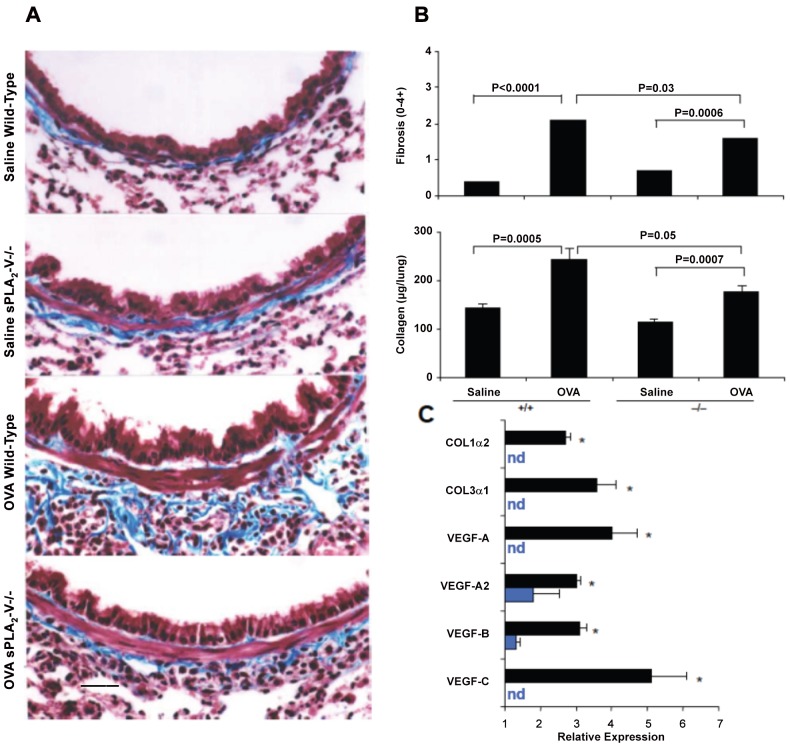
Impaired chronic allergen-induced airway remodeling and collagen deposition in sPLA_2_-V^−/−^ mice. Lung tissue was obtained on d 76 from sPLA_2_-V^+/+^ and sPLA_2_-V^−/−^ mice treated with either saline or OVA. **A.** Sections underwent Masson's trichrome staining. Scale bar, 50 μm. Micrographs are representative from 4–5 mice per group. **B.** Airway subepithelial fibrosis (0–4+ scale) was determined by morphometry, and lung collagen deposition (μg/lung) was determined by Sircol™ assay (*n* = 4–5, each group). **C.** Collagen (COL1α2 and COL3α1) and VEGF (VEGF-A, VEGF-A2, VEGF-B, and VEGF-C) gene expression in OVA-treated sPLA_2_-V^+/+^ (black bars) and sPLA_2_-V^−/−^ (blue bars) mice was determined by qPCR; nd  =  not detected in samples from the sPLA_2_-V^−/−^ mice. *P<0.05 OVA-treated sPLA_2_-V^+/+^ versus sPLA_2_-V^−/−^ mice (*n* = 4–5, each group).

The effect of sPLA_2_-V deficiency on allergen-induced persistent infiltration of lung tissue by eosinophils and other inflammatory cells and airway goblet cell metaplasia, subepithelial fibrosis, and collagen and VEGF gene expression was examined in a chronic asthma model of lung remodeling (**Figures** **4** **–** **7**). On d 76, sPLA_2_-V^+/+^ mice had a dense infiltrate in the lung interstitium of eosinophils and other inflammatory cells (**Figure** **4** **)** and increase in airway goblet cells (**Figure** **5**). By morphometric analysis, the total inflammatory cell and eosinophil infiltration was reduced by 61% (*P* = 0.01) and 67% (*P* = 0.001) respectively and the goblet cell metaplasia was diminished by 58% (*P* = 0.02) in OVA-treated sPLA_2_-V^−/−^ mice compared to wild-type controls (**Figure** **6**). After long-term OVA challenge, the sPLA_2_-V^+/+^ mice had increased deposition of subepithelial collagen and increased lung collagen content compared to saline-treated controls (**Figure** **7A,B**). The subepithelial fibrosis and increased collagen content observed in OVA-treated wild-type mice were modestly reduced by 24% (*P* = 0.03) and 31% (*P* = 0.05) respectively in the allergen-challenged sPLA_2_-V^−/−^ mice (**Figure** **7B**). By quantitative real-time PCR (qPCR), the OVA-treated sPLA_2_-V^−/−^ mice had marked impairment in collagen (i.e., COL1α2 and COL3α1), and VEGF (i.e., VEGF-A, VEGF-A2, VEGF-B, and VEGF-C) gene expression in their lungs compared to OVA-treated wild-type controls (**Figure** **7C**).

### Effect of sPLA_2_-V Deficiency on Th2 Cytokine and DC Responses

**Figure 8:**
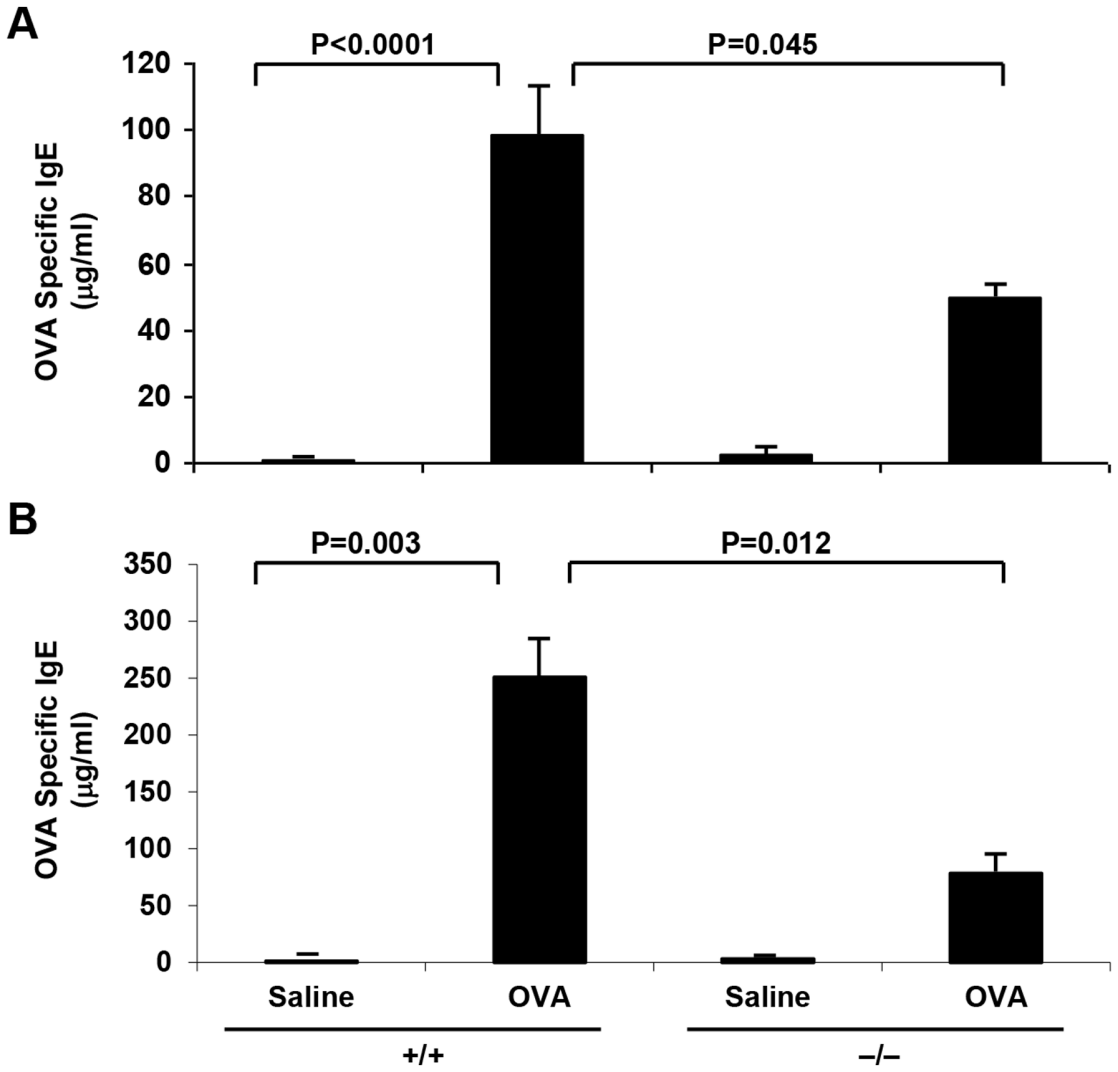
Reduced IgE levels in OVA-treated sPLA_2_-V^−/−^ mice. OVA-specific IgE levels were determined in plasma obtained on d 23 (**A**) and d 76 (**B**) from saline (*Saline*)- and OVA (*OVA*)-treated sPLA_2_-V^+/+^ (+/+) and sPLA_2_-V^−/−^ (−/−) mice (*n* = 4–5, each group).

**Figure 9:**
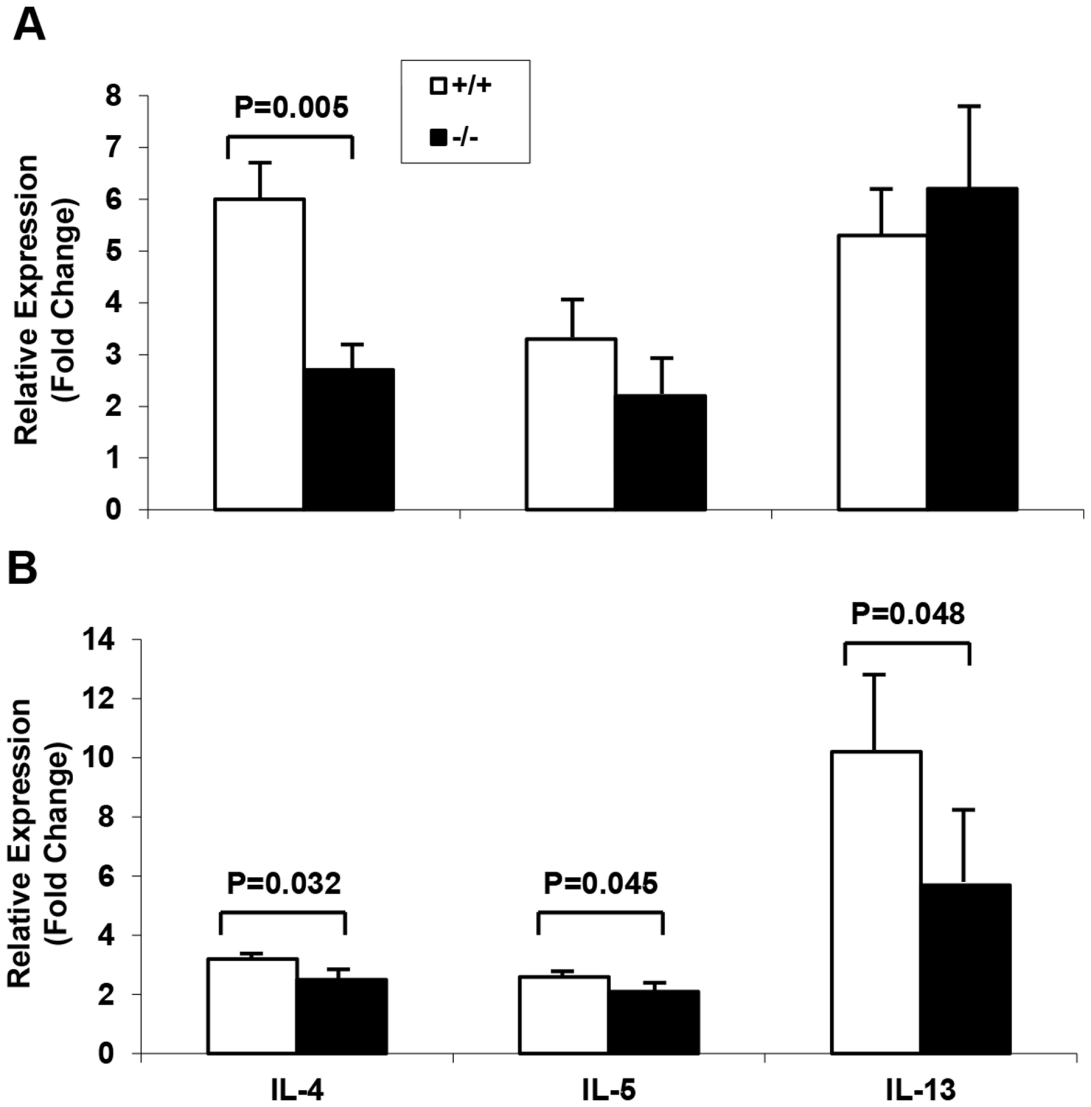
Decreased lung Th2 cytokine gene expression in OVA-treated sPLA_2_-V^-/-^ mice. Gene expression of Th2 cytokines IL-4, IL-5, and IL-13 in whole lung tissue from sPLA_2_-V^+/+^ and sPLA_2_-V^−/−^ mice obtained on d 23 (**A**) and d 76 (**B**) was determined by qPCR (*n* = 4–5, each group).

The effect of sPLA_2_-V-deficiency on Th2 cytokine responses was next examined. On d 23 and d 76, circulating levels of OVA-specific IgE in blood were decreased by 50% (*P* = 0.045, **Figure** **8A**) and 71% (*P* = 0.012, **Figure** **8B** **)** respectively in sPLA_2_-V^−/−^ mice compared to wild-type mice after OVA sensitization and challenge. Pulmonary expression of Th2 cytokines IL-4, IL-5, and IL-13 in lung tissue of sPLA_2_-V^−/−^ and sPLA_2_-V^+/+^ mice was determined by qPCR (**Figure** **9**). Gene expression of IL-4, IL-5, and IL-13 on d 23 (**Figure** **9A**) and d 76 (**Figure** **9B**) was increased in whole lung tissue of OVA-treated sPLA_2_-V^+/+^ mice, compared to saline-treated controls. On d 23, IL-4 expression was decreased 55% (*P* = 0.005) in OVA-treated sPLA_2_-V^−/−^ mice, compared to wild-type controls; Il-5 and IL-13 levels were not statistically different between the OVA-treated sPLA_2_-V^−/−^ and sPLA_2_-V^+/+^ mice (**Figure** **9A**). On d 76, IL-4, IL-5, and IL-13 gene expression was decreased 22% (*P* = 0.032), 19% (*P* = 0.045), and 49% (*P* = 0.048) respectively in OVA-treated sPLA_2_-V^−/−^ mice, compared to sPLA_2_-V^+/+^ mice (**Figure** **9B**).

**Figure 10:**
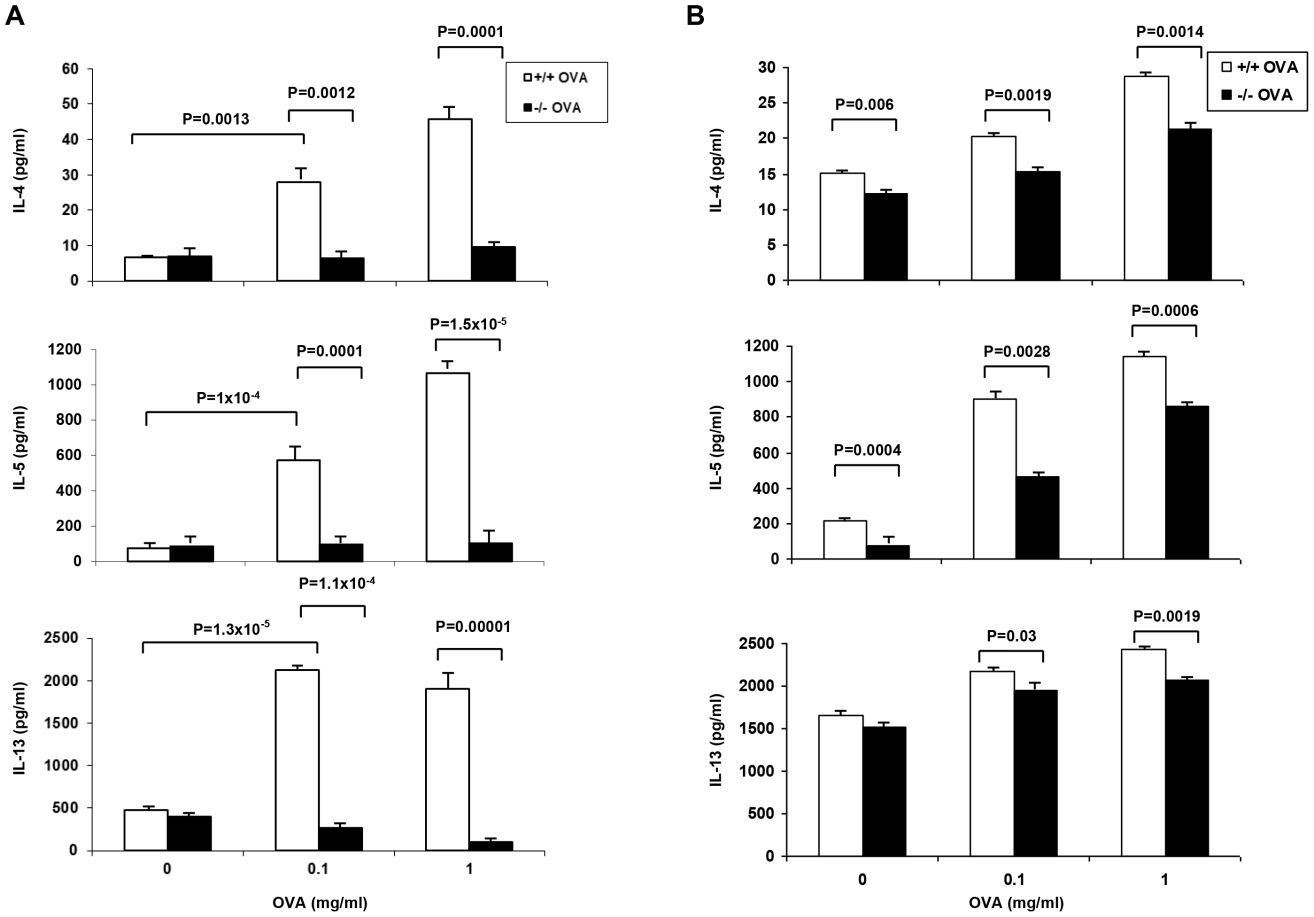
sPLA_2_-V deficiency impairs Th2 cytokine production in lung lymph node cells and splenocytes. On d 76, lung lymph node cells (**A**) and total splenic cells (**B**) from OVA-sensitized/challenged sPLA_2_-V^−/−^ mice and wild-type controls were incubated with 0.1 or 1 mg/ml OVA or medium alone for 72 h and supernatants collected for EIA assay of IL-4, IL-5, and IL-13 (*n* = 4–5, each group).

**Figure 11:**
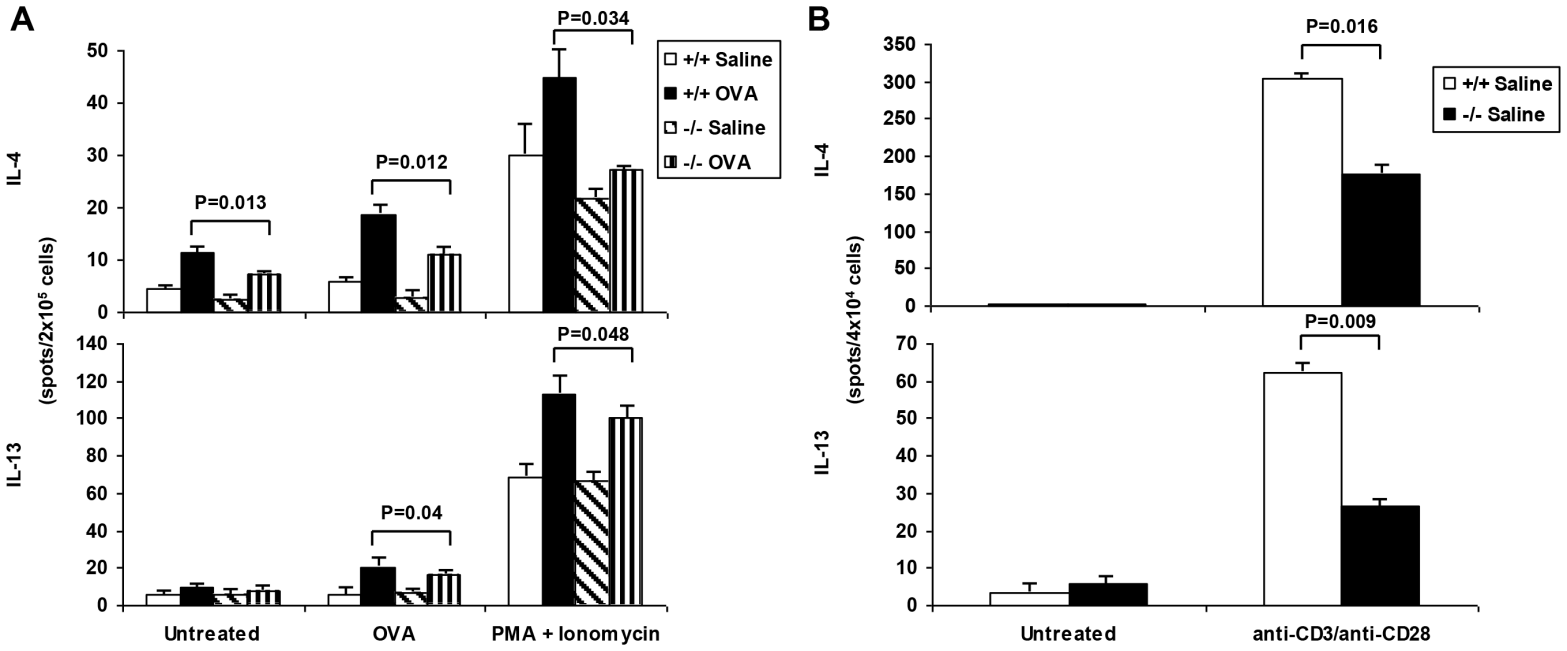
Single cell immunospot assay of IL-4 and IL-13 production by splenocytes and splenic CD4^+^ T cells from sPLA_2_-V^−/−^ mice. A. Elispot assay of IL-4 and IL-13 production by splenocytes obtained on d 76 from sPLA_2_-V^+/+^ mice treated with saline (+/+ *Saline*) or OVA (+/+ *OVA*) and sPLA_2_-V^−/−^ mice treated with saline (^−^/^−^ *Saline*) or OVA (−/− *OVA*) and incubated in the absence (*Untreated*) or presence of either 500 μg/ml OVA (*OVA*) or 5 ng/ml PMA/500 ng/ml ionomycin (*PMA + Ionomycin*) (*n* = 4-5, each group). **B.** Elispot assay of IL-4 and IL-13 production by CD4^+^ T cells isolated from the total splenic cells obtained on d 76 from sPLA_2_-V^+/+^ mice incubated in the absence (*Untreated*) or presence of anti-CD3/anti-CD28 antibodies (*anti-CD3/anti-CD28*).

The chronic asthma model was chosen for more detailed analyses of the nature of this relative impairment in Th2 cytokine responses in the sPLA_2_-V^−/−^ mice. On d 76, Th2 cytokine responses of lung lymph node cells, splenocytes, and splenic CD4^+^ T cells were determined in OVA-treated sPLA_2_-V^−/−^ mice compared to wild-type controls (**Figures** **10** **and** **11**). Three different strategies were employed to assess the Th2 responses – antigen (i.e., OVA)-specific stimulation, protein kinase C activation (i.e., PMA plus ionomycin), and mimicking of T cell receptor (TCR) activation (i.e., anti-CD3/anti-CD28 stimulation). Lung lymph nodes were collected from the sPLA_2_-V^−/−^ and sPLA_2_-V^+/+^ mice and stimulated with OVA *in vitro* (**Figure** **10A**). The production of IL-4, IL-5, and IL-13 was significantly increased in the OVA-treated lung lymph node cells of the sPLA_2_-V^+/+^ mice compared to saline-treated controls (**Figure** **10A**). OVA-induced release of Th2 cytokines was impaired in the lung lymph node cells of the sPLA_2_-V^−/−^ mice. *In vitro* production of IL-4, IL-5, and IL-13 proteins by OVA-treated lung lymph node cells obtained from sPLA_2_-V^−/−^ mice treated with 0.1 mg.ml OVA was decreased by 77% (*P* = 0.012), 83% (*P* = 0.0001), and 87% (*P* = 1.1×10^−4^) respectively and 1 mg/ml OVA was reduced by 78% (*P* = 0.0001), 90% (*P* = 1.5×10^−5^), and 95% (*P* = 0.00001) respectively compared to wild-type controls (**Figure** **10A**). *In vitro* production of IL-4, IL-5, and IL-13 proteins by OVA-treated total spleen cells obtained from sPLA_2_-V^−/−^ mice treated with 0.1 mg.ml OVA was decreased by 24% (*P* = 0.019), 48% (*P* = 0.028), and 10% (*P* = 0.03) respectively and 1 mg/ml OVA was reduced by 26% (*P* = 0.014), 25% (*P* = 0.0006), and 15% (*P* = 0.019) respectively compared to wild-type controls (**Figure** **10B**). Next, OVA- and PMA/ionomycin-stimulated production of IL-4 and IL-13 by splenic cells was determined by a single cell immunospot assay (ELISPOT). The production of IL-4 and IL-13 by splenocytes of sPLA_2_-V^−/−^ mice in response to OVA was decreased by 42% (*P* = 0.012) and 19% (*P* = 0.04) respectively and in response to PMA + ionomycin was reduced by 39% (*P* = 0.034) and 11% (*P* = 0.048) respectively, in comparison to wild-type controls (**Figure** **11A**). In response to anti-CD3/anti-CD28 stimulation (**Figure 11B** **),** IL-4 and IL-13 production by splenic CD4^+^ T cells isolated from sPLA_2_-V^−/−^ mice was decreased by 42% (*P* = 0.016) and 57% (*P* = 0.009) respectively compared to controls.

**Figure 12:**
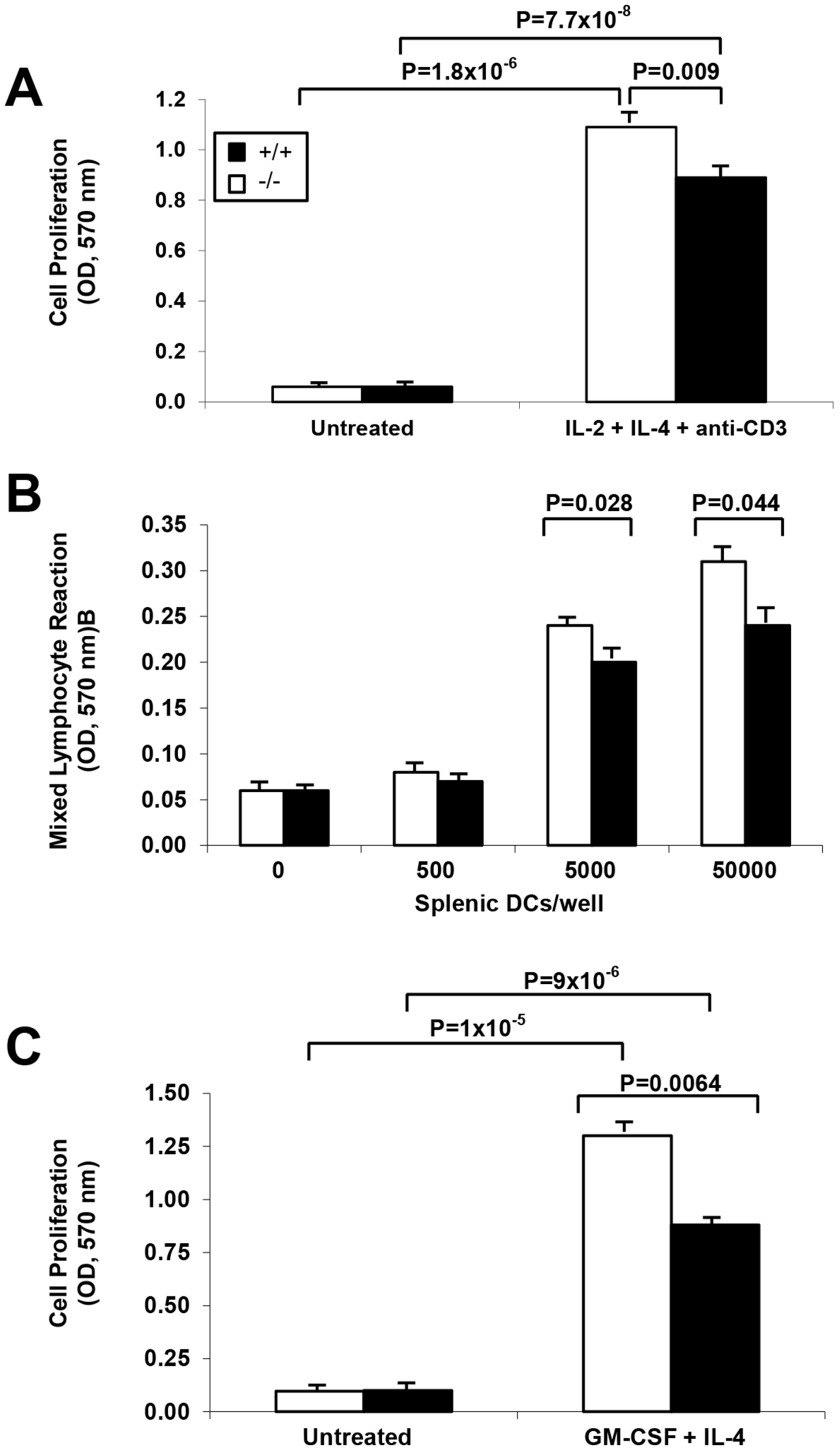
Effect of sPLA_2_-V deficiency on CD4^+^ T cell and DC proliferation. **A.** Cell proliferation of wild-type and sPLA_2_-V^−/−^ splenic CD4^+^ T cells cultured in the absence (*Untreated*) or presence of IL-2 and IL-4 in anti-CD3-coated plates (*IL-2 + IL-4 + anti-CD3*) for 72 h was determined by MTT assay (*n* = 4–5, each group). **B.** Allogeneic CD4+ cells from C57Bl6 mice were cultured with irradiated (3000 rad) splenic DCs (0–50000 cells/well) from wild-type or sPLA2-V−/− mice for 72 h with cell proliferation measured by MTT assay (*n* = 4–5, each group). **C.** BMDC proliferation was assessed by MTT assay on d 9 after 3 days in culture in the absence (*Untreated*) or presence of GM-CSF and IL-4 (*GM-CSF + IL-4*) (*n* = 4–5, each group).

**Figure 13:**
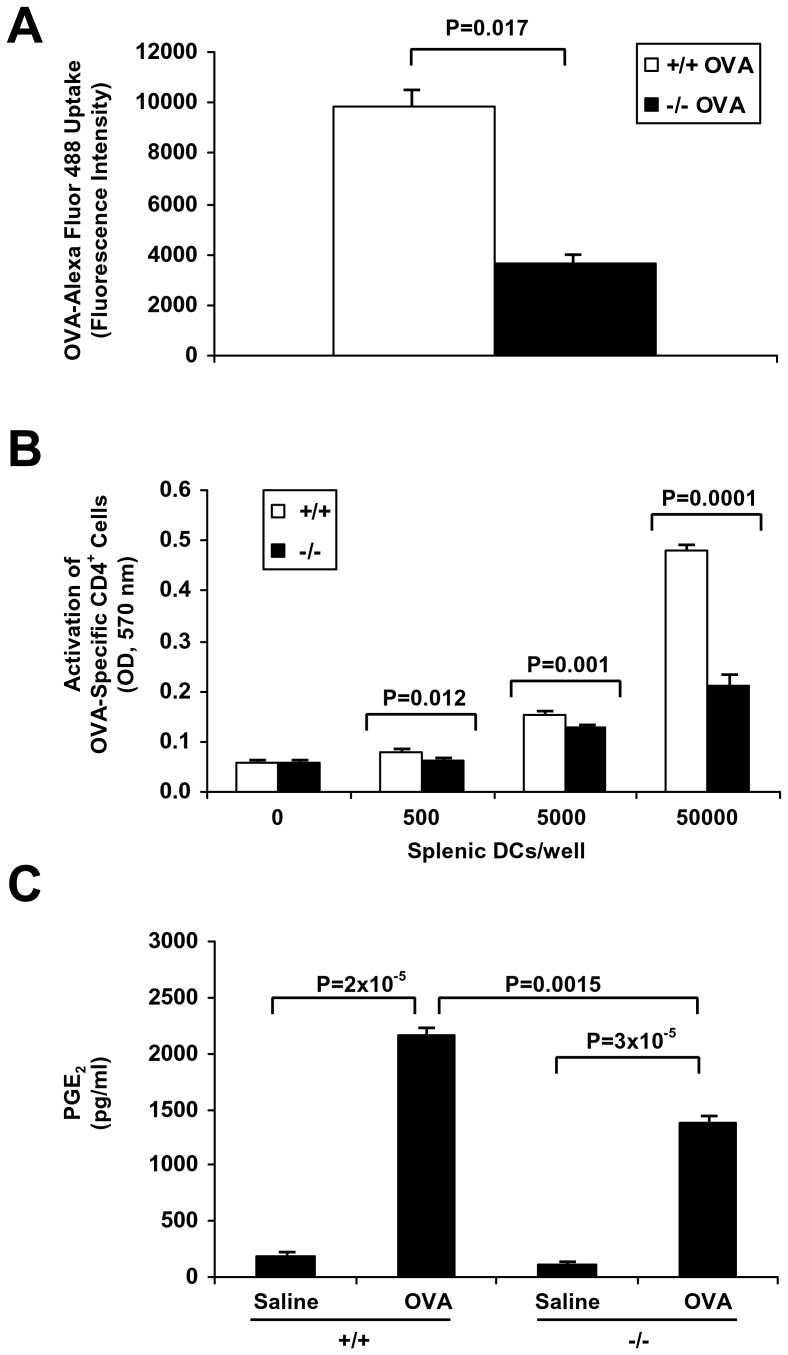
Effect of sPLA_2_-V deficiency on DC OVA uptake, presentation to OVA-transgenic T cells, and PGE_2_ production. **A.** Endocytosis of Alexa Fluor 488-labeled OVA (0.1 mg/ml) by BMDCs from sPLA_2_-V^+/+^ (+/+) and sPLA_2_-V^−/−^ (−/−) mice was assessed at 2 h by flow cytometry using a BD FACSCanto™ Flow Cytometry System with the mean fluorescence intensity (MFI) representing the amount of incorporated tracer by APC-CD11c+ cells (*n* = 4–5, each group). **B.** The antigen-presenting activity of splenic DCs in sPLA2-V-deficient mice in comparison to wild-type controls was assessed using CD4^+^ T cells carrying the MHC class II restricted rearranged T cell receptor (TCR) transgene, Tg(DO11.10)10Dlo that react to OVA peptide antigen. Irradiated (3000 rad) splenic DCs from sPLA_2_-V^+/+^ (+/+) and sPLA_2_-V^−/−^ (−/−) mice were cultured overnight with OVA (1 mg/ml) at 37°C in 5% CO2; the splenic DCs (0–50000 DCs/well) were then incubated with CD4+ naïve T cells (1×106 T cells/well) isolated from OVA-TCR transgenic mice for 48 h in the absence or presence of OVA323-339 peptide (1 μg/ml). **C.** PGE2 production by BMDCs was assayed by EIA on d 7 in culture after incubation for 24 h with OVA (1 mg/ml) (*n* = 4–5, each group).

To understand the nature of the Th2 cytokine defect in the sPLA_2_-V-deficient mice, CD4^+^ T cell and DC proliferation, and DC antigen processing and eicosanoid production was studied (**Figures** **12** and **13**). Although both sPLA_2_-V-deficient and wild-type splenic CD4^+^ T cells had a marked increase in proliferation under Th2 polarizing conditions in response to IL-2 and IL-4 after culture in anti-CD3-coated plates *in vitro*, the magnitude of this response was slightly decreased by 19% (*P* = 0.009) in the sPLA_2_-V-deficient mice (**Figure** **12A**). The mixed lymphocyte reaction (MLR) cell proliferative response was compared between sPLA_2_-V^−/−^ and sPLA_2_-V^+/+^ splenic DCs. Under basal conditions, increasing numbers (0–50000 DCs per well containing 2×10^5^ allogeneic CD4^+^ T cells) of DCs from wild-type mice led to a marked increase in cell proliferation that was modestly reduced by 12% (*P* = 0.028) and 21% (*P* = 0.044) with 5000 and 50000 sPLA_2_-V^−/−^ DCs/well respectively (**Figure** **12B**). The effect of sPLA_2_-V-deficiency on proliferation of BMDCs in culture with GM-CSF and IL-4 was studied. At baseline, no difference in proliferation was observed between the sPLA_2_-V^−/−^ and sPLA_2_-V^+/+^ BMDCs (**Figure** **12C**). In contrast, proliferation of the sPLA_2_-V-deficient BMDCs was reduced by 33% (*P* = 0.0064) compared to wild-type controls by d 9 in culture (**Figure** **12C**).

Endocytosis of Alexa Fluor 488-labeled OVA by BMDCs was examined in sPLA_2_-V-deficient mice and wild-type controls. Uptake of Alexa Fluor 488-labeled OVA by sPLA_2_-V^−/−^ BMDCs was reduced by 63% (*P* = 0.017) compared to sPLA_2_-V^+/+^ BMDCs (**Figure** **13A**). We next compared the antigen-presenting activity of DCs from wild-type versus sPLA_2_-V-deficient mice using CD4^+^ T cells carrying the MHC class II restricted rearranged T cell receptor (TCR) transgene, Tg(DO11.10)10Dlo that react to OVA peptide antigen (**Figure** **13B**). The ability of sPLA_2_-V^−/−^ splenic DCs to activate OVA-specific CD4^+^ T cells (at the ratio 50000 splenic DCs/well containing 1x10^6^ CD4^+^ naïve T cells) was decreased by 55% (*P* = 0.0001) compared to wild-type controls (**Figure** **13B**).

The effect of sPLA_2_-V deficiency on BMDC-derived eicosanoid production was determined. OVA (1 mg/ml) did not induce a significant increase in the 5-LO arachidonic acid products LTB_4_ or CysLTs by either the sPLA_2_-V^+/+^ or sPLA_2_-V^−/−^ BMDCs compared to saline-treated controls (data not shown). In contrast, OVA triggered a marked release of PGE_2_ by sPLA_2_-V^+/+^ BMDCs that was significantly increased over the levels of saline-treated controls (**Figure** **13C**). This increased release of PGE_2_ induced by OVA was reduced by 36% (*P* = 0.0015) in BDMCs of sPLA_2_-V^−/−^ mice (**Figure** **13C**).

## Discussion

We have previously shown that disruption of the gene for sPLA_2_-X in mice leads to a dramatic reduction in airway inflammation, remodeling, and hyperresponsiveness in a mouse model of airway asthma [12]. Given the large number of sPLA_2_s in the mammalian genome, it seems prudent to examine the involvement of other sPLA_2_s in an asthma model. Because sPLA_2_-V and sPLA_2_-X sPLA_2_ are very active at hydrolyzing the membranes of mammalian cells, these two enzymes have been our first priority in genetic disruptions studies of sPLA_2_s in complex disease models. In prior work, Munoz et al reported a study of airway inflammation in OVA-administered sPLA_2_-V knockout mice [19]. Their observations are consistent with what we find in our independent study. In their and our (**Figure** **3**) studies, sPLA_2_-V expression is upregulated after OVA sensitization, and the protein is found in airway epithelium, mononuclear cells, and smooth muscle cells. Both studies report that loss of sPLA_2_-V leads to a reduction in inflammatory cell infiltration into the airways in response to OVA. (i.e., the influx of total inflammatory cells and eosinophils, was decreased by 45% and 57% respectively in the Munoz study [19] and 59% and 54% respectively in the present report (**Figure 1A**). Similar to Munoz et al. [19], we also found that sPLA2-V-deficiency impaired allergen-induced airway hyperresponsiveness to methacholine (**Figure 1B**).

The study by Munoz et al. [19], did not examine the effect of group V sPLA_2_-deficiency on allergen-induced airway remodeling (i.e., goblet cell metaplasia and subepithelial fibrosis) and provided little mechanistic insight (i.e., arachidonic acid metabolism and T cell/dendritic cell function was not examined) into the mechanism(s) by which group V sPLA_2_ regulates allergic pulmonary inflammation. Our study thus adds the important dimension that a drop in airway inflammation in this model due to sPLA_2_-V deletion is likely due to a reduction in the primary immune response as reflected by changes in eicosanoid and Th2 cytokine generation.

Giannattasio et al. [20] used house dust mite *Dermatophagoides farinae* as antigen to induce pulmonary inflammation without systemic immunization in a mouse asthma model to explore the effect of sPLA_2_-V deficiency on the adaptive immune response. In this dust mite asthma model, they observed a greater reduction in eosinophil infiltration into the BAL fluid and goblet cell metaplasia (95% and 80% reductions respectively) than we did in the OVA model (54% and 58% reductions respectively) compared to wild-type controls. We also showed impairment in collagen and VEGF gene expression and modest reductions in lung collagen deposition in the OVA model (**Figure** **7**), effects not examined in the Giannattasio study [20]. Whereas, no differences in eicosanoid production were observed between sPLA_2_-V^−/−^ and sPLA_2_-V^+/+^ mice in the dust mite asthma model, a moderate reduction in eicosanoids (30% decrease in CysLTs and 45% decrease in PGD_2_) was seen in the BAL fluid of OVA-treated sPLA_2_-V^−/−^ mice compared to wild-type controls (**Figure** **2**). In both the *D. farinae* and OVA (**Figure** **8**) asthma models, antigen-specific IgE levels were reduced in the sPLA_2_-V-deficient mice compared to controls.

In the dust mite asthma model, Giannattasio et al. [20] observed significant reductions in total lung IL-5 and IL-13 transcripts in the sPLA_2_-V^−/−^ knockouts and decreased Il-4, IL-5, and IL-13 Th2 cytokine levels from pulmonary lymph node cells after *ex vivo* restimulation with *D. farinae* [20]. In the acute asthma model, we found a 55% reduction in IL-4 transcripts in the OVA-treated sPLA_2_-X knockout mice but no decrease in IL-5 or IL-13 transcripts. In contrast, in the chronic asthma model (**Figure** **9B**), each of the Th2 cytokine transcripts was modestly reduced in the sPLA_2_-deficient mice (i.e., reductions of 22% for IL-4, 19% for IL-5, an 49% for IL-13). Similar to the Giannattasio study [20], restimulation of lung lymph node cells with OVA allergen *ex vivo* in the chronic OVA model led to marked reductions in IL-4, IL-5, and IL-13 production (**Figure** **10A**) with lesser reductions in these Th2 cytokines by OVA-restimulated spleen cells (**Figure** **10B**). We also observed moderate impairment of sPLA_2_-V-deficient splenic T cell production of IL-4, IL-5, and IL-13 cytokines after *ex vivo* allergen-specific, protein kinase C, and TCR activation (**Figure** **11**) and a small reduction in the ability of sPLA_2_-V-deficient splenic CD4^+^ T cells to proliferate under Th2 polarizing conditions (**Figure** **12A**). Our data and that of Giannattasio et al. [20] suggest that the reduced airway inflammation in the OVA-driven sPLA_2_-V knockout mouse compared to the wild-type controls can be explained by a decrease in the primary immune response, leading to lower levels of OVA-specific IgE as well as Th2 cytokines. IL-4 induces class switching and release of IgE by B cells, and IL-5 plays an important role in the induction of the eosinophil influx into the lungs in allergen-driven models of asthma [21], [22]. IL-13 is a key mucus secretagogue and pro-fibrotic cytokine that causes fibroblast proliferation and collagen deposition in the airways [23]. IL-13 is also the primary Th2 cytokine in induction of airway hyperresponsiveness [24].

At this point, the role of sPLA_2_-V in augmenting the primary immune response is becoming better understood. Prior studies have demonstrated that peritoneal macrophages from sPLA_2_-V^−/−^ mice phagocytose zymosan particles significantly less well than wild-type mice [25], clearance of immune complexes is reduced in sPLA_2_-V^−/−^ mice [26], and that the presence of sPLA_2_-V in dendritic cells is key for cell maturation and antigen processing in dust mite-induced lung inflammation [20] suggesting that perhaps sPLA_2_-V deficiency may affect processing of allergen by dendritic cells. CysLTs have important actions on DC maturation and function. CysLTs augment the antigen-presenting capacity of dendritic cells in the lung [27]. LTC_4_, but not LTD_4_ or LTB_4_, matures DCs in a superior fashion than PGE_2_ to stimulate DC-driven CD4^+^ T cell responses and antigen-specific T cell induction [28]. CysLTs also increase the capacity of BMDCs to induce Th2 immune responses in the lungs after adoptive transfer in mice [29]. CysLTs promote the migration of dendritic cells to lymph nodes [30]. Pretreatment of asthmatics with a CysLT_1_ receptor antagonist reduces the allergen-induced decrease in circulating CD33^+^ DCs indicating a role for CysLTs in the trafficking of myeloid DCs *in vivo* [31]. LTB_4_ through its BLT_1_ [32] and BLT_2_ [33] receptors also promotes DC migration. CysLT-mediated activation and chemotaxis of monocyte-derived immature dendritic cells is inhibited by the immunoregulatory cytokine IL-10 suggesting a link between IL-10 regulatory responses and the 5-LO pathway [34]. Human DCs differentially express the CysLT receptors CysLT_1_ and CysLT_2_ depending upon maturation signals such as the Toll-like receptor (TRL) 4 agonist lipopolysaccharide (LPS) [35]. The TRL2 agonist zymosan down-regulates CysLT_1_ receptor expression on human monocyte-derived DCs diminishing their responsiveness to LTD_4_ [36]. In the *D. farinae* mouse asthma model, CysLT_2_ receptor negatively regulates CysLT_1_ receptor activation of BMDCs and also the expression of the CysLT_1_ receptor on the surface of these DCs suggesting that a competitive balance between these two CysLT receptors may regulate allergic lung inflammation [37].

In this report, we also found that BMDCs from sPLA_2_-V-deficient mice have decreased proliferation after stimulation. The sPLA_2_-V^−/−^ DCs exhibit defects in their uptake of OVA and ability to activate CD4^+^ cell proliferation and present OVA to OVA-transgenic T cells. These defects in DC function are associated with impairment in PGE_2_ production by these immunoregulatory cells. PGE_2_ has key effects on cytokine production by antigen-presenting cells such as DCs and T cells affecting DC and T helper cell differentiation and effector actions [38]–[40]. PGE_2_ promotes DC maturation, activation, and migration [41]. Recent studies have shown that PGE_2_ signaling through its EP1 and EP3 receptors is needed for optimal survival of DC progenitors and DC development *in vivo* via regulation of the receptor tyrosine kinase Flt3 on the DC progenitor cells [42]. Thus, impairment in eicosanoid generation (both 5-LO and COX arachidonate metabolites) in sPLA_2_-V^−/−^ mice may lead to a diminution in both Th2 and DC responses in the airways. With the development of selective sPLA_2_ inhibitors [12], blockade of group V sPLA_2_ may provide a novel therapeutic opportunity in the treatment of asthma and other allergic disorders.

## Materials and Methods

### Ethics Statement

All animal use procedures were approved by the IACUC of the University of Washington (Animal Welfare Assurance No. A346401).

### Allergen Challenge in Mice

Homozygous sPLA_2_-V^−/−^ mice were generated as previously described [18], and the genotype was verified by PCR. Acute- and chronic-term mouse asthma model protocols were employed. In the acute asthma model, sPLA_2_-V^−/−^ C57Bl6 mice and their wild-type sPLA_2_-V^+/+^ littermates were immunized by intraperitoneal (i.p.) injection with 10 μg OVA (Pierce Biotechnology, Inc., Rockford, IL) and 1.125 mg alum (Sigma-Aldrich Corporation, St. Louis, MO) in 0.2 ml normal saline on d 0, 7, and 14 and exposed to 1% aerosolized OVA [43] for 40 min on d 21, 22, and 23 [12]. Control groups received 0.2 ml normal saline with alum i.p. on d 0, 7, and 14, and saline by aerosol on d 21, 22, and 23. In the chronic asthma model [44], [45], mice received an i.p. injection of 100 μg of OVA (0.2 ml of 0.5 mg/ml) complexed with alum on d 0 and 14. Mice received an intranasal (i.n.) dose of 100 μg OVA (0.05 ml of 2 mg/ml) on d 14, and 50 μg OVA (0.05 ml of 1 mg/ml) on d 26, 27, 28, 47, 61, 73, 74, and 75. Control groups received 0.2 ml normal saline with alum i.p. on d 0 and 14 and 0.05 ml saline without alum i.n. on d 14, 26, 27, 28, 47, 61, 73, 74, and 75.

### Pulmonary Function Testing

On d 23 (40 min after the last aerosol challenge with OVA or saline in the acute model), invasive pulmonary mechanics were measured in mice in response to methacholine as described [12]. Mice received aerosolized solutions of methacholine (0, 3.125, 6.25, 12.5, 25, and 50 mg/ml in normal saline) with R_L_ determined from measures of pressure and flow and expressed as cm H_2_O/ml/s using a Model PLY4111 plethysmography system (Buxco Research Systems, Wilmington, NC).

### BAL Fluid and Blood Collection

After completion of plethysmography on d 23, the left lung was tied off at the mainstem bronchus, and the right lung lavaged three times with 0.5 ml of normal saline. After centrifugation at 250×*g* for 5 min at 4°C, total BAL fluid cells were counted with eosinophils stained with 0.05% eosin [46]. BAL fluid eicosanoid analyses were performed only in mice that did not get the invasive R_L_ measurements; the supernatant was processed for eicosanoid assays as described below. Lung tissue was collected for qPCR assay of Th2 cytokines. Plasma samples were obtained on d 23 and assayed for OVA-specific IgE.

### Eicosanoid Analyses

For CysLTs and PGE_2_ analyses, BAL fluid supernatant was processed on solid-phase extraction cartridges followed by detection using enzyme immunoassay (EIA) kits from Cayman Chemical Company (Ann Arbor, MI) as described [12]. PGD_2_ was analyzed using a 0.25 ml aliquot of the BAL fluid supernatant using the PGD_2_-MOX EIA kit (Cayman Chemical Company) [12].

### Lung Tissue, Paratracheal Lymph Node, and Spleen Collection

On d 76 (24 h after the last i.n. dose of OVA or saline, chronic model), the lungs were collected for histopathology, collagen, and qPCR analyses. Paratracheal lymph nodes and spleens were collected for cytokine analyses and isolation of spleen cells, CD4^+^ T cells, and DCs as described below. Plasma samples were also obtained on d 76 and assayed for OVA-specific IgE.

### Lung Histopathology

The upper and lower lobes of the left lung were collected and 5 mm sections prepared [46]. Ten airways (0.4–0.7 mm in diameter and surrounded by smooth muscle cells) per mouse were randomly selected for morphometric analysis by individuals blinded to the protocol design [46]. The sections were stained with hematoxylin and eosin (H&E) to determine total inflammatory cell infiltration [46] on a semi-quantitative scale (0–4+), and eosinophil number per unit lung tissue area (2,200 μm^2^) [47], [48]. Alcian blue staining was used to identify airway goblet cells.

### Immunocytochemistry

sPLA_2_-V expression in mouse lung was determined by immunocytochemistry using light microscopy [49]. The sections were incubated with the primary antibody, polyclonal rabbit anti-sPLA_2_-V-specific antisera [50] at a 1:50 dilution for 25 min at room temperature followed by rinsing in PBS and incubation with the secondary antibody, goat anti-rabbit antibody conjugated to horseradish peroxidase (Vector Laboratories, Burlingame, CA) at a 1:20 dilution for 25 min. As controls, PBS or normal rabbit IgG (Vector Laboratories) were used in place of the primary antibody. To detect peroxidase, the sections were incubated with 0.5% 3′, 3′-diaminobenzidine tetrachloride (Sigma-Aldrich Corporation) in PBS and 0.15% hydrogen peroxide for 15 min at room temperature; nuclei were counterstained with 1% methyl green in distilled water for 3 min.

### Collagen Assay

Collagen content of the right lung was determined by the Sircol™ collagen assay (Biocolor Ltd., Newtownabbey, Northern Ireland, UK).

### OVA-specific IgE Assay

For assay of OVA-specific IgE, Nunc 96-well flat bottomed plates (Nalge Nunc International, Rochester, NY) were coated with OVA (50 μg/ml) in 1X PBS overnight at room temperature, washed 3 times with 1X PBS containing 0.05% Tween-20, and blocked with 1X PBS containing 3% BSA for 60 min at room temperature. 50 μl plasma samples (1:1 in 1X PBS) or varying concentrations of internal standard (Clone C38-2 anti-mouse IgE, BD Biosciences, San Diego, CA) were added per well and incubated for 90 min at 37°C, and washed/blotted dry. After addition of 100 μl (1:100 in 1X PBS) biotin-conjugated rat anti-mouse IgE monoclonal antibody (Clone R35–72; BD Biosciences) to each well, the plates were incubated overnight at 4°C; then 100 μl Streptavidin-HRP-conjugated secondary antibody (1:1000 in 1X PBS, BD Biosciences) was added per well and plates incubated at 37°C for 90 min. 100 μl of 2,2′-azinobis(3-ethylbenzthiazoline-sulfonic acid (i.e., one tablet dissolved in 100 ml of 0.05 M phosphate-citrate buffer, pH 5.0 and 25 μl 30% H_2_O_2_; Sigma-Aldrich Corporation) substrate solution was added per well and after incubation for 30 min at room temperature, the plates were read at OD 405 nm with a standard curve constructed by linear regression analysis of the absorbances in comparison to serial dilutions of known concentrations of mouse IgE.

### qPCR

Total RNA was isolated from the right lung using an RNeasy mini kit (QIAGEN Inc., Valencia, CA), and mRNA levels for IL-4, IL-5, IL-13, COL1α2, COL3α1, vascular endothelial growth factor (VEGF)-A, VEGF-A2, VEGF-B, VEGF-C, and GAPDH determined by qPCR using a model 7900HT Fast Real-Time PCR System [Applied Biosystems (ABI), Foster City, CA] as described [12]. PCR DNA sizes were ∼100 bp and confirmed by gel electrophoresis.

### Spleen Cell and CD4^+^ T Cell Isolation

Spleens were placed in RPMI-1640 with 25 mmol/L HEPES (Invitrogen Corporation, Carlsbad, CA) supplemented with 10% (vol/vol) heat-inactivated fetal bovine serum (FBS; Invitrogen), cut into small pieces with scissors, and strained through a 70 μm BD Falcon^TM^ cell strainer (BD Biosciences, San Jose, CA) to create single-cell suspensions. Red cells were lysed using BD PharmLyse™ lysing buffer (BD Biosciences). CD4^+^ T cells were purified from splenic lymphocytes by magnetic depletion of B cells, macrophages, DCs, NK cells, granulocytes, erythroid precursors, and CD8^+^ T cells using MACS® CD4^+^ T Cell Isolation Kit (Miltenyi Biotec Inc., Auburn, CA).

### Th2 Cytokine Analyses

For Th2 cytokine production by lymph node and spleen cells after *in vitro* restimulation with OVA, paratracheal lymph node and spleen cells from OVA-treated sPLA_2_-V^−/−^ mice and wild-type controls were cultured at a density of 2×10^6^ per well in 96-well tissure culture plate in RPMI-1640 medium with 10% FBS containing OVA (0.1 and 1 mg/ml) or medium alone for 72 h at 37°C in 5% CO_2_/95% air at 37°C. IL-4, IL-5, and IL-13 levels in the supernatants were determined by EIA kits (eBioscience, San Diego, CA). For enzyme-linked IL-4 immunospot (ELISPOT) (BD Biosciences) and IL-13 ELISPOT (R&D Systems, Minneapolis, MN) assays, total spleen cells from OVA- and saline-treated sPLA_2_-V^−/−^ mice and wild-type controls were collected, plated in 96-well plates (2×10^5^ cells/well), and incubated in triplicate in 5% CO_2_/95% air at 37°C in the absence or presence of phorbol myristate acetate (PMA, 5 ng/ml, Sigma-Aldrich Corporation) and ionomycin (500 ng/ml, Sigma-Aldrich Corporation) for 24 h or OVA (500 μg/ml) for 5 d according to the manufacturer's protocol (BD Biosciences). To assess CD4^+^ T cell Th2 cytokine production, splenic CD4^+^ T cells (4×10^4^ cells/well) were incubated in triplicate in 5% CO_2_/95% air at 37°C in the absence or presence of hamster anti-mouse CD3ε (1 μg/ml)/hamster anti-mouse CD28 (2 μg/ml) monoclonal antibodies (BD Biosciences) for 5 d and IL-4 and IL-13 immunospot assays performed.

### CD4^+^ T Cell Proliferation

4×10^5^ purified CD4^+^ T cells isolated from sPLA_2_-V^−/−^ and sPLA_2_-V^+/+^ mice were cultured in complete RPMI medium with 50 ng/ml recombinant mouse IL-4 and 10 ng/ml recombinant mouse IL-2 in 96-well plates coated with 10 mg/ml anti-CD3 (BD Biosciences) for 3 d at 37°C in 5% CO_2_ using the Cayman Chemical Co. MTT [3-(4,5-dimethylthiazol-2-yl)-2,5-diphenyltetrazolium bromide] Proliferation Assay Kit to assess cell proliferation.

### Spleen DC Purification

Spleens were placed in 3 ml RPMI with 1 mg/ml DNase (Worthington Biochemical Corporation, Lakewood, NJ) and 50 mg/ml collagenase (Worthington Biochemical Corporation) in 6-well plates. 200 μl of this RPMI was injected in each spleen that was then cut into small pieces and strained through a 70 μm cell strainer (BD Biosciences). Red cells were lysed using BD PharmLyse™ lysing buffer (BD Biosciences). After washing with AutoMACS^TM^ Rinsing solution (Miltenyi Biotec Inc.), DCs were purified with CD11cMicroBeads® (Miltenyi Biotec Inc.) [51].

### MLR

2×10^5^ allogeneic CD4^+^ cells from C57Bl6 mice were cultured with irradiated (3000 rad) splenic DCs (0–50000 cells/well) from wild-type or sPLA_2_-V^−/−^ mice in complete RPMI-1640 at 37°C in 5% CO_2_ for 72 h. The MTT assay was used to determine cell proliferation [52].

### Stimulatory Activities of DCs Against Antigen-specific T Cells

The antigen-presenting function of the DCs from the sPLA_2_-V^−/−^ mice in comparison to wild-type controls was examined employing CD4^+^ T cells carrying the MHC class II restricted rearranged T cell receptor (TCR) transgene, Tg(DO11.10)10Dlo that react to OVA peptide antigen, then secrete Th2 cytokines and proliferate [52]. Irradiated (3000 rad) splenic DCs from wild-type or sPLA_2_-V^−/−^ mice were cultured with OVA (1 mg/ml) overnight at 37°C in 5% CO_2_. The splenic DCs (0–50000 DCs/well) were cultured with 1×10^6^ CD4^+^ naïve T cells isolated from BALB/c OVA-TCR transgenic mice (C.Cg-Tg(DO11.10)10Dlo/J transgenic mice**;** The Jackson Laboratory, Bar Harbor, ME) for 48 h at 37°C in 5% CO_2_ with or without OVA323–339 peptide that binds to I-A(d) major histocompatibility complex (MHC) class II protein (1 μg/ml; InvivoGen, San Diego, CA).

### BMDC Culture

Murine DCs were generated from bone marrow of sPLA_2_-V^−/−^ mice and wild-type mice. Briefly, bone marrow was harvested by flushing femurs and tibias with PBS containing 1% FBS. Cells were resuspended at 2×10^6^ cells/ml in GIBCO® RPMI-1640 (Invitrogen Corporation) supplemented with 5 ng/ml of recombinant mouse GM-CSF (R&D Systems, Inc., Minneapolis, MN) and 10 ng/ml of recombinant mouse IL-4 (BD Biosciences). On d 3 and 5 of culture, half of the medium was replaced with fresh medium containing GM-CSF and IL-4. On d 6, loosely adherent cells were harvested and DCs purified with CD11c MicroBeads® according to the manufacturer's instructions (Miltenyi Biotec Inc.) [52].

### BMDC Cell Proliferation

BMDC cells from d 0, 3, and 6 in culture were cultured in triplicate at 2×10^5^ cells per well in 96-well plates for 3 d in complete GIBCO® RPMI-1640 (Invitrogen Corporation) supplemented with 5 ng/ml of recombinant mouse GM-CSF (R&D Systems, Inc.) and 10 ng/ml of recombinant mouse IL-4 (BD Biosciences) at 37°C in 5% CO_2_ and assayed for cell proliferation using the MTT cell proliferation kit [51].

### BMDC Cell OVA-Alexa Fluor 488 Phagocytosis

OVA-Alexa Fluor 488 (Invitrogen Corporation) was added to 1×10^6^ BMDCs at a final concentration of 0.1 mg/ml. Endocytosis of the tracer was halted at 2 h by rapid cooling of the cells on ice and BMDC cells washed with ice-cold HBSS. The fluorescence intensity of the cells was analyzed by flow cytometry (FACSCanto™ Flow Cytometry System, BD Biosciences). Incubation of cells with endocytic tracer on ice was used as background control. The mean fluorescence intensity (MFI) represented the amount of incorporated tracer by APC-CD11c^+^ cells (eBioscience) [53].

### OVA-induced BMDC Cell Eicosanoid Release

1×10^6^ purified d 6 BMDCs in complete GIBCO® RPMI-1640 (Invitrogen Corporation) were cultured with OVA (1 mg/ml) overnight with supernatants collected for assay of PGE_2_, LTB_4_, and CysLTs using EIA kits from Cayman Chemical Co.

### Statistical Analysis

The data are reported as the mean ± SE of the mean. Differences were analyzed for significance (*p*<0.05) by analysis of variance (ANOVA) using the least significant difference method.
